# Recycling Rusty Iron with Natural Zeolite Heulandite to Create a Unique Nanocatalyst for Green Hydrogen Production

**DOI:** 10.3390/nano11123445

**Published:** 2021-12-20

**Authors:** Mohamed Shaban, Mohammad BinSabt, Ashour M. Ahmed, Fatma Mohamed

**Affiliations:** 1 Department of Physics, Faculty of Science, Islamic University in Madinah, Al-Madinah Al-Munawarah 42351, Saudi Arabia; 2 Nanophotonics and Applications (NPA) Lab, Physics Department, Faculty of Science, Beni-Suef University, Beni-Suef 62514, Egypt; ashour.elshemey@gmail.com (A.M.A.); f_chem2010@yahoo.com (F.M.); 3 Chemistry Department, Faculty of Science, Kuwait University, P.O. Box 5969, Safat 13060, Kuwait; Mohammad.binsabt@ku.edu.kw; 4 Polymer Research Laboratory, Chemistry Department, Faculty of Science, Beni-Suef University, Beni-Suef 62514, Egypt

**Keywords:** rusted iron, Fe_2_O_3_/zeolite nanocomposite, water splitting, hydrogen production, photocatalyst

## Abstract

Corrosion-induced iron rust causes severe danger, pollution, and economic problems. In this work, nanopowders of Fe_2_O_3_ and Fe_2_O_3_/zeolite are synthesized for the first time using rusted iron waste and natural zeolite heulandite by chemical precipitation. The chemical composition, nanomorphologies, structural parameters, and optical behaviors are investigated using different techniques. The Fe_2_O_3_/zeolite nanocomposite showed smaller sizes and greater light absorption capability in visible light than Fe_2_O_3_ nanopowder. The XRD pattern shows crystalline hematite (α-Fe_2_O_3_) with a rhombohedral structure. The crystallite sizes for the plane (104) of the Fe_2_O_3_ and Fe_2_O_3_/zeolite are 64.84 and 56.53 nm, respectively. The Fe_2_O_3_ and Fe_2_O_3_/zeolite have indirect bandgap values of 1.87 and 1.91 eV and direct bandgap values of 2.04 and 2.07 eV, respectively. Fe_2_O_3_ and Fe_2_O_3_/zeolite nanophotocatalysts are used for solar photoelectrochemical (PEC) hydrogen production. The Fe_2_O_3_/zeolite exhibits a PEC catalytic hydrogen production rate of 154.45 mmol/g.h @ 1 V in 0.9 M KOH solution, which is the highest value yet for Fe_2_O_3_-based photocatalysts. The photocurrent density of Fe_2_O_3_/zeolite is almost two times that of Fe_2_O_3_ catalyst, and the IPCE (incident photon-to-current conversion efficiency) reached ~27.34%@307 nm and 1 V. The electrochemical surface area (ECSA) values for Fe_2_O_3_ and Fe_2_O_3_/zeolite photocatalysts were 7.414 and 21.236 m^2^/g, respectively. The rate of hydrogen production for Fe_2_O_3_/zeolite was 154.44 mmol h^−1^/g. This nanophotocatalyst has a very low PEC corrosion rate of 7.6 pm/year; it can retain ~97% of its initial performance. Therefore, the present research can be applied industrially as a cost-effective technique to address two issues at once by producing solar hydrogen fuel and recycling the rusted iron wires.

## 1. Introduction

Fossil fuel burning is the major source of CO_x_ emissions (CO_2_ and CO) in atmospheric air, which causes global warming. The resulting air pollution can have catastrophic effects on humans and animals alike [1,2]. Hydrogen fuel is a carbon-free, renewable, and environmentally friendly source of energy that can be used as an ideal alternative to fossil fuels.

Therefore, the developments of effective techniques for large hydrogen fuel production at reasonable cost are important research areas. The photoelectrochemical (PEC) hydrogen production utilizing semiconductor-based catalysts is a promising technique to meet these requirements. In the PEC process, the photocatalyst produces an electron/hole pair after absorbing a photon, which is then isolated, transported, and contributed to the cathodic hydrogen evolution/anodic oxygen evolution reactions at applied voltage [3,4]. Under incident light with a suitable wavelength, electron/hole pairs are created in the semiconductor. The holes reacted with H_2_O to generate hydroxyl radical (OH). The electrons can react with O_2_ to produce superoxide radicals ($O_{2}^{-}$). These reactive species are primarily responsible for the water splitting and hydrogen production [5]. There are several semiconductor materials such as WO_3_, ZrO_2_, In_2_O_3_, SnO_2_, Fe_2_O_3_, TiO_2_, ZnO, CuO, and CdS that were applied to upgrade the PEC performance. Among them, Fe_2_O_3_ is used as a photocatalyst for the PEC due to its hard solubility, high chemical stability, low cost, and massive abundance [6,7]. Additionally, it is a non-toxic and ecologically benign substance, all of which are required for large-scale solar energy conversion at a reasonable cost. Fe_2_O_3_ has semiconducting properties with a narrow bandgap (∼2.1 eV). This low bandgap enables it to be a good photocatalyst in the visible region. However, this material has many drawbacks that limit its application in practical photocatalytic such as low diffusion lengths of holes, poor conductivity, fast electron–hole recombination, poor adsorption property, agglomeration, and difficulty in being recovered [8]. Several studies immobilized the Fe_2_O_3_ nanoparticles on different supports, such as activated carbon, silica, alumina, clay, and zeolite to overcome these disadvantages. Among them, zeolite is of particular interest because, besides its semiconducting nature, it has a high adsorption capacity against organic contaminants. Zeolite possesses ionic exchange properties that are idyllic for the adsorption/degradation of organic dyes [9,10]. It also has enormous unique areas, adjustable hydrophobicity/hydrophilicity, and photochemical stability [11,12]. In addition, zeolite is low-cost, abundant, and bio-compatible. Zeolite is a monocrystal mineral composed of Si and Al atoms in a tetrahedral arrangement (TO_4_; T = Si, Al) [13]. It can be used in many applications such as cement, porcelain, electronics, and water splitting for the production of hydrogen. When a semiconductor is supported on a suitable support, such as zeolites, the semiconductor particles are evenly dispersed, preventing them from aggregating. 

In the past few years, zeolite was used as a support for semiconductor-based PEC catalysts to enhance the hydrogen production rate. The ZnCo/CdS/zeolite heterostructure was prepared and optimized by Jia-Hui et al. to achieve photocatalytic hydrogen activity 59 times greater than that of pristine CdS, which is ascribed to zeolite’s role in improving the separation and transportation capacity of photo-generated charge carriers [14]. Yue and Khan reported the formation of vacant sites on the zeolite surface due to the exchange of ions in titano-zeolites, which assists the hydrogen photoproduction [15]. Additionally, Pt/zeolite and Cu/zeolite were prepared and applied for the hydrogen [16,17]. Owing to its large use in many applications, iron has been considered one of the primary manufacturing materials over the past decades. Iron corrosion happens after the iron contacts the air moisture. The corrosion of iron structures causes millions of tons of rusty waste to form, resulting in danger, environmental pollution, and economic issues. Therefore, considering the worldwide vast use of iron wires, the recycling/reuse of rusty waste is predicted to substantially decrease the wastes amounts, leading to the creation of recycling-oriented societies.

Hence, the Fe_2_O_3_ nanoparticles production from rusted iron wastes can thus be considered in many fields as a viable alternative to synthetic and natural iron supplies. Previously, different techniques have been used to prepare Fe_2_O_3_ nanostructures such as sol-gel, spray pyrolysis, hydrothermal, chemical vapor deposition, and thermal evaporation [18,19]. Most of these methods require complicated reactions, high energy intakes, and poor product yield. Since no special additives or equipment are needed, chemical precipitation is considered the most effective and low-cost technique for the production of Fe_2_O_3_. 

The objective of this work is to replace the iron precursors with rust wastes as a source of iron for the synthesis of Fe_2_O_3_ and Fe_2_O_3_/zeolite nanopowder by chemical precipitation. The prepared Fe_2_O_3_/zeolite nanocomposite is applied for the PEC production of solar hydrogen fuel. The photon-to-electron and photon-to-hydrogen conversion efficiencies are calculated for Fe_2_O_3_ and Fe_2_O_3_/zeolite. 

## 2. Materials and Experimental Procedures

### 2.1. Materials

Natural zeolite was delivered from a zeolite mine located southwest of Taiz (Al-Ahyuq region, Taiz City, Yemen). HCl and KOH were received from El-Nasr Company (Cairo, Egypt). All chemicals were at least 99 percent pure, and they were utilized just as they were bought, with no further purification. Rusted iron wires were collected from construction sites.

### 2.2. Preparation of the Zeolite, Fe_2_O_3_ and Fe_2_O_3_/Zeolite

Rusted iron wire fragments were collected from construction sites in Egypt’s Beni-Suef City. The average length of wires is about 30 cm with a diameter of about 1 cm. The color of the wires is dark red. Upon cutting to small fragments, the rusted wires were washed using deionized (DI) water. A total of 10 g of these pieces was dissolved in 80 mL of HCl (37%) and 170 mL DI water under magnetic stirring at 85 °C. The solution was filtered, and 20 mL of H_2_O_2_ (30%) was added to the obtained pale green-colored solution. Under intense 60 min-stirring, the ammonia solution was dropped to the iron solution. In a glass beaker with a volume of 200 mL, the iron was precipitated. Varying volumes of ammonium hydroxide solution (10, 15, and 20 mL) were used to prepare Fe_2_O_3_ powders with different crystallite sizes. The samples were labeled as Fe_2_O_3_ (I), Fe_2_O_3_ (II), and Fe_2_O_3_ (III), where I, II, and III refer to the 10, 15, and 20 mL of ammonia supplied to the reaction, respectively. Then, the resulting precipitated iron powder was filtrated before washing and drying. Then, the collected powder was heated for 3 h at 500 °C. A total of 15 g of raw zeolite mine was washed with DI water and dried in the air. Then, it was triggered mechanically by ball milling. Table 1 shows the conditions for ball milling parameters.

**Figure 1:**
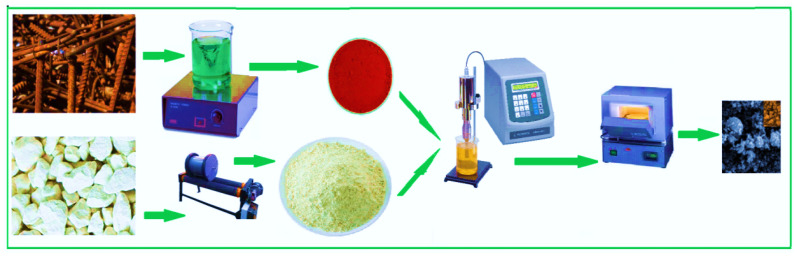
Schematic of the synthesis steps of Fe_2_O_3_/zeolite.

For preparing Fe_2_O_3_/zeolite nanocomposite with optimized composition, different weight ratios of activated zeolite and iron powder (Fe_2_O_3_ (III)) were added to 100 mL of DI water under ultrasonication for 3 h. The total weight of Fe_2_O_3_/zeolite nanocomposite is kept at 2 g. The weight ratios were 0.2/1.8, 0.6/1.4, 0.8/1.2, 1.0/1.0, 1.2/0.8, 1.4/0.6, and 1.8/0.2. The resulting mixtures were dried at 80 °C for 12 h. Finally, the Fe_2_O_3_/zeolite nanocomposites were calcinated at 550 °C for 240 min. The nanopowders were recorded as xFe_2_O_3_/yzeolite, where x and y were denoted to the adding weight of Fe_2_O_3_ and zeolite, respectively. The synthesis steps of Fe_2_O_3_/zeolite nanocomposite are illustrated by a schematic in Figure 1.

### 2.3. Characterizations

A Philips X’Pert Pro MRD diffractometer (XRD, λ = 0.154 nm, Philips X’Pert Pro MRD, Royston, UK) was utilized to obtain the X-ray diffraction (XRD) patterns of the samples with an operating voltage of 40 kV in the range from 5° to 80°. The samples nanomorphologies were examined using a JEOL JSM-5400LV scanning electron microscope (SEM, JEOL, Tokyo, Japan). The chemical compositions were investigated by Energy Dispersive X-ray spectrometry (EDX, JEOL JED-2300 SEM, Tokyo, Japan). FT-IR (Fourier transform-infrared) spectra of Fe_2_O_3_ and Fe_2_O_3_/zeolite nanocomposite were examined through Vertex 70 FTIR-FT Raman spectrometer (Billerica, MA, USA). The UV/Vis optical properties of the samples were scanned in the range 250–900 nm with an increment of 1 nm by UV-Vis double beam spectrophotometer (LAMBDA 950, PerkinElmer Inc., Waltham, MA, USA). About 0.05 g nanopowder is dispersed in 10 mL of dimethylformamide by ultrasonic for 3 h. Then, 3 mL of the prepared suspension is used for UV-Vis spectroscopy scanning in a standard quartz cuvette.

### 2.4. PEC Water Splitting Measurements

The PEC behaviors in 0.9 M KOH (100 mL, pH 13.5) were measured at room temperature (20 °C) utilizing a Keithly measuring-source unit (Tektronix Company, model: 2400, Beaverton, OR, USA) with LabTracer software and a 400 W metal-halide lamp (New-port, 66926-500HX-R07, Newport, UK) with a set of linear optical filters (307–636 nm). The sweeping scan rate was 1 mV/s. Fe_2_O_3_ and Fe_2_O_3_/zeolite doses of 1 g were used. The PEC current density–voltage (J–V) curves were quantified in darkness, monochromatic, and white light exposure conditions. In addition, the Fe_2_O_3_/zeolite stability was investigated using current density–time (J–t) measurements. All PEC measurements were carried out in a quartz cell of volume 150 mL.

## 3. Results and Discussion

### 3.1. Photocatalysts Characterization

#### 3.1.1. Structural of Fe_2_O_3_ and Fe_2_O_3_/Zeolite

**Figure 2:**
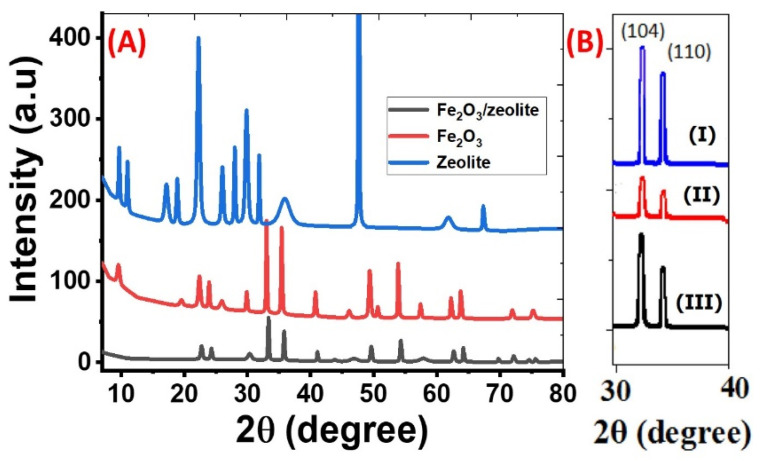
(**A**) XRD patterns of zeolite, Fe_2_O_3_, and Fe_2_O_3_/zeolite nanocomposite; and (**B**) XRD (104) and (110) peaks of Fe_2_O_3_ (I), Fe_2_O_3_ (II), and Fe_2_O_3_ (III).

The crystallinity and phase of the Fe_2_O_3_, zeolite, and Fe_2_O_3_/zeolite nanocomposite were identified using XRD analysis as seen in Figure 2A. Zeolite’s distinctive XRD peaks, in Figure 2A, are noted at 2θ ~9.68°, 11.00°, 17.16°, 18.87°, 22.24°, 26.01°, 27.97, 29.84°, 31.83°, 35.88°, 47.58°, 61.76°, and 67.31°. Such peaks correspond to the crystallographic plane (020), (200), (111), (−131), (−222), (−422), (−351), (−530), (−202), (005), (311), and (223), based on PDF card No. 00-053-1176, respectively. Based on the XRD card, the type of zeolite is heulandite.

For iron oxide, the XRD pattern in Figure 2A suggests that crystalline hematite (α-Fe_2_O_3_) with rhombohedral structure (space group: R-3c) was formed according to the standard card No. 01-089-0597. This agrees with the previously reported data for Fe_2_O_3_ [20]. The pattern of Fe_2_O_3_ nanoparticles displays the core α-Fe_2_O_3_ feature peaks. These peaks are found at 33.00°, 35.39°, 49.32°, 53.84°, and 63.74° and correspond to the planes (104), (110), (024), (116), and (300). The sharp and intensive peaks indicate the high purity and crystallinity of the synthesized hematite nanoparticles using bulk Fe-based rust. These XRD data are similar to previously synthesized iron oxide in many works using synthetic precursors [21,22,23]. From the estimated FWHM of the strongest (104) and (110), the crystallite sizes of the Fe_2_O_3_ nanoparticles were estimated based on the Debye–Scherrer relation to be ~64.84 and 50.46 nm, respectively.

For zeolite, many distinct peaks are observed at 22.72° (101), 41.05° (210), and 54.24° (221), corresponding to tetragonal zeolite (Al_0.05_Si_0.95_O_2_) according to card No. 04-002-8520. As illustrated in Figure 2A, the main core features of XRD patterns of Fe_2_O_3_ and Fe_2_O_3_/zeolite are very close, indicating that the introduction of zeolite did not affect the structural properties of the Fe_2_O_3_ photocatalyst. However, the coupling of Fe_2_O_3_ with zeolite leads to an increase in the FWHM and a slight shift in the plane position of the Fe_2_O_3_ toward higher angles after coupling. Hence, the crystallites sizes of (104) and (110) peaks for Fe_2_O_3_ nanoparticles were decreased to 56.53 and 47.85 nm for Fe_2_O_3_/zeolite nanocomposite. Similar behavior was reported for hydrothermally prepared 4A-zeolite supported alpha-Fe_2_O_3_ [24]. In addition, the relative intensities of the diffraction peaks of Fe_2_O_3_/zeolite nanocomposite became weaker than the peaks of Fe_2_O_3_, indicating a change in the crystallinity of the photocatalyst due to the distribution of Fe_2_O_3_ on the surface of the zeolite [25]. The structural parameters such as crystallite size (D), interplanar distance (d), dislocation density (δ), and microstrain (ε) are calculated for the highest two peaks, (104) and (110), utilizing the XRD patterns of Fe_2_O_3_ and Fe_2_O_3_/zeolite nanopowders. Besides peak position, peak height, and relative intensity, the obtained values are displayed in Table 2. For the two planes (104) and (110), the value of microstrain increases while d-spacing decreases after loading the zeolite with Fe_2_O_3_. The strongest peak corresponds to the plane (104), which indicates the preferred growth orientation of hematite. This growth orientation is beneficial to carrier transport [26]. The number of lattice defects was estimated depending on the dislocation density, δ, which refers to the dislocation lines length per unit volume of the crystal. The δ value is estimated using the relation; δ = 1/D^2^. The values of δ for the Fe_2_O_3_ and Fe_2_O_3_/zeolite at the preferred orientation (104) are 2.378 × 10^−4^ and 3.129 × 10^−4^ dislocation/nm^2^, respectively. The increase in dislocation density proposes the decrease of Fe_2_O_3_/zeolite crystallinity [27], which strongly influences the photocatalytic properties of the fabricated nanomaterials. This is also confirmed by the decreasing of the XRD peaks intensities after loading Fe_2_O_3_ on zeolite, as seen in Table 2. The existence of a high density of the defects in the Fe_2_O_3_/zeolite nanocrystallites can contribute positively to the photocatalytic properties as a result of the active surface area increase and the formation of a high density of the active centers [28]. These active centers may result from the formation of static charge fields about the dislocation lines [29].

Figure 2B shows the XRD (104) and (110) peaks of Fe_2_O_3_ (I), Fe_2_O_3_ (II), and Fe_2_O_3_ (III) that were prepared using different amounts of ammonium hydroxide solution (10, 15, and 20 mL). From Figure 2B, the average crystallite size (D) for the highest two planes (104) and (110) were calculated by the Debye–Scherer equation at different amounts of ammonia solution. The average values of D for Fe_2_O_3_ (I), Fe_2_O_3_ (II), and Fe_2_O_3_ (III) are found to be 57.65, 44.12, and 36.42 nm respectively. Then, the average crystallite size of Fe_2_O_3_ depends on the volume of used ammonium hydroxide. According to the effective mass model, when particle size is reduced at the nanoscale, quantum confinement has an influence on electrons in nanoparticles. Changing the quantum (crystallite) size can alter the optical characteristics. As a result, the crystallite size is critical to the generation of hydrogen.

#### 3.1.2. Surface Morphology

**Figure 3:**
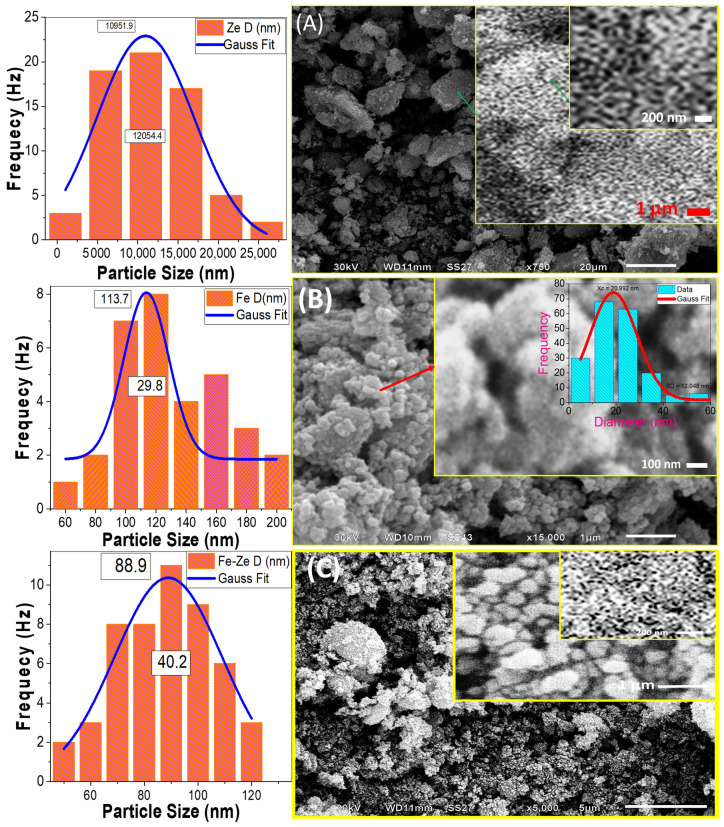
SEM micrographs and the corresponding particle size distribution for (**A**) natural zeolite, (**B**) Fe_2_O_3_, and (**C**) Fe_2_O_3_/zeolite. The inset of (**B**) shows the pore diameter distribution.

It is well-known that the photocatalytic activity of the photocatalyst is strongly related to its surface morphology. The morphologies of natural zeolite, Fe_2_O_3_, and Fe_2_O_3_/zeolite nanopowders are examined utilizing the SEM technique as shown in Figure 3.

**Figure 4:**
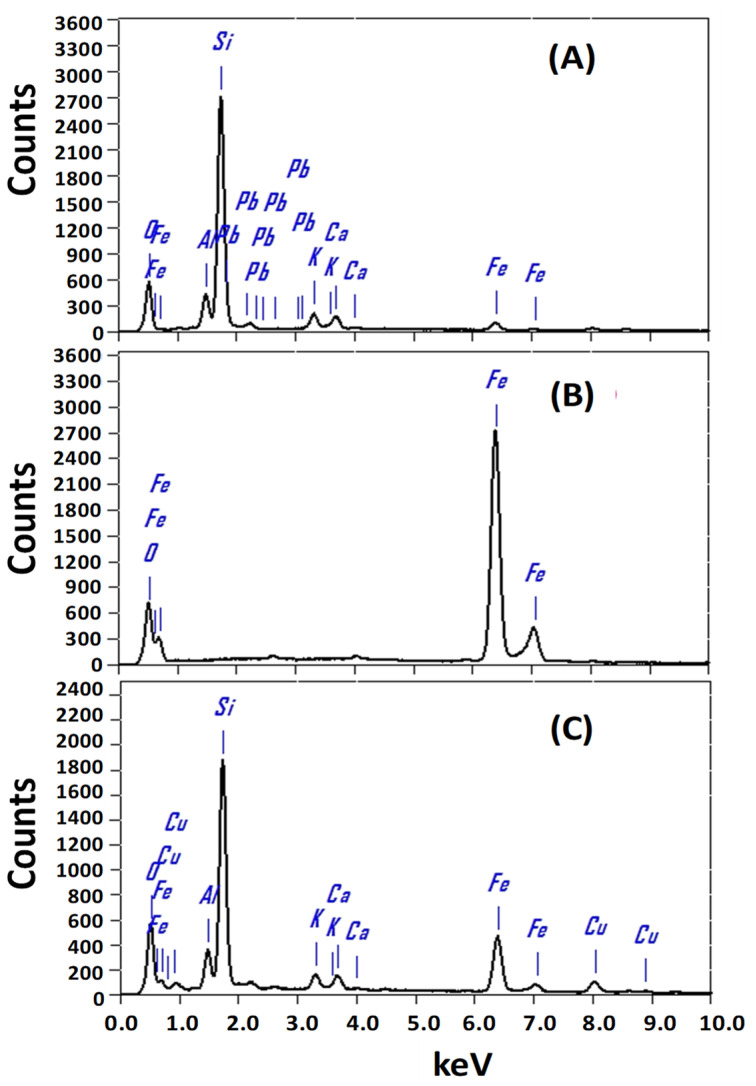
EDX spectrum of (**A**) zeolite, (**B**) Fe_2_O_3_, and (**C**) Fe_2_O_3_/zeolite nanocomposite.

The SEM images of natural zeolite, Figure 3A, show micro/nano-stones in nonuniform shapes of various sizes. The sizes of stones for zeolite are changed from 21.6 to 3.2 µm, as seen in the corresponding particle size distribution (left of Figure 4A) of the particle size distribution. The mean particle size is 10.951 ± 0.820 μm with a standard deviation of 6.027 ± 1.647 μm. A close look at the image reveals the existence of many small nanoprotrusions/nanograins over zeolite particle surfaces with an average size of ~115 nm. Additionally, there are many small nanopores with a diameter of ~71 nm on the surface of zeolite with irregular shapes as seen in high magnification Figure 3A. The high surface area due to the porous framework provides a chance to incorporate iron oxide nanoclusters inside the pore cavity of zeolite [30]. Additionally, these pores can adsorb organic pollutants, which can increase photodegradation efficiency.

The Fe_2_O_3_ nanopowder was composed of many nanoparticles with semi-spherical shapes. The SEM image of Fe_2_O_3_ nanoparticles shows that the nanoparticles are small in size, seen in Figure 3B. The corresponding particle size distribution is shown on the left of Figure 3B. Based on Gaussian fitting; the mean size of Fe_2_O_3_ nanoparticle is 113.65 ± 4.67 nm with a standard deviation of 14.92 ± 5.95 nm. These nanoparticles are self-assembled and aggregated to form nanopores of average diameter ~20.99 nm with a standard deviation of ±6.02 nm, as shown from the inset pore-diameter distribution of Figure 3B.

Fine spherical Fe_2_O_3_ nanoparticles coated the zeolite surface and appeared as homogeneous distributions that produced a nano-sized Fe_2_O_3_ coating surface over zeolite stones after loading zeolite with the intended Fe_2_O_3_ photo-catalyst, Figure 3C. It is also possible that the Fe_2_O_3_ coating was quite homogeneous, with no obvious uncoated zeolite sites. The size of the Fe_2_O_3_ nanoparticles seems to be decreased after loading on zeolite compared to the free-standing Fe_2_O_3_ nanopowder. The size distribution of the supported Fe_2_O_3_ nanoparticles on the surface of zeolite, left of Figure 3C, indicates an average value of 88.94 ± 1.67 nm. Additionally, the high magnification SEM image, inset of Figure 3C, shows a more homogeneous pore-diameter distribution with a mean value of 35.50 ± 2.25 nm.

The interlock between Fe_2_O_3_ nanoparticles and their precipitation over the zeolite is expected to be beneficial for PEC activity. Haileyesus et al. reported that similar interlock structures can offer a rapid migration of the induced electrons and holes to the catalyst surface, which leads to a low probability of recombination [31]. Additionally, the decrease of the particle size to the nanoscale and the widening of the pores can offer a huge effective surface area of Fe_2_O_3_ nanocatalyst. This can offer intensive absorption of the incident light.

#### 3.1.3. Chemical Compositions of the Photocatalysts

To identify the chemical compositions of the designed photocatalysts and atomic ratios of the elements, the EDX spectra of zeolite, Fe_2_O_3_, and Fe_2_O_3_/zeolite nanocomposite were measured and presented in Figure 4. The chemical composition for the zeolite shows the main three elements (O, Al, and Si) as revealed by EDX analysis. Additionally, small signals for K, Ca, and Fe are observed, in addition to a small trace from Cu. These signals are similar to previously reported signals for the zeolite [32].

The EDX analysis of Fe_2_O_3_, Figure 4B, indicated the presence of O (37.62%) and Fe (62.38%) signals as the main components at around 0.525 and 6.398 keV. The atomic ratios of Fe to O suit the stoichiometry ratios of Fe_2_O_3_ well. This confirms the high purity of the prepared Fe_2_O_3_ nanopowder, which coincides with the XRD results. After loading Fe_2_O_3_ onto zeolite, there are main four characteristic peaks for O, Al, Si, and Fe with atomic ratios of 53.12%, 6.30%, 26.63%, and 9.01%, respectively. This indicates the successive loading of Fe_2_O_3_ onto the surface of the zeolite.

#### 3.1.4. The Photocatalysts’ Optical Properties

**Figure 5:**
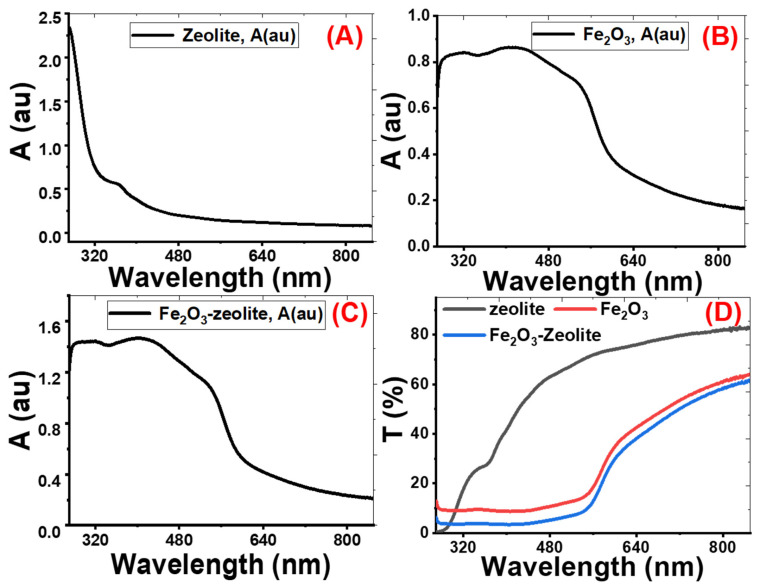
Absorbance (A%) for zeolite (**A**), Fe_2_O_3_ (**B**), and Fe_2_O_3_/zeolite (**C**), and transmittance (T%) for all samples (**D**).

Nanomaterials’ optical properties are important characteristics that influence their uses [33,34]. The absorption (A) and transmittance (T) spectra from 250 to 850 nm of zeolite, Fe_2_O_3_, and Fe_2_O_3_/zeolite are shown in Figure 5. The zeolite sample has a sharp peak corresponding to a strong absorption band at the UV region (below λ = 300 nm), as seen in Figure 5A. Then, the absorbance decreases sharply with increasing the wavelength from 280 up to 850 nm. Therefore, the zeolite sample displayed a very low spectral response in the visible region.

The absorbance spectra for Fe_2_O_3_ and Fe_2_O_3_/zeolite show similar optical behaviors, as seen in Figure 5B,C. The Fe_2_O_3_ has strong photoabsorption in the UV and visible spectral regions [35]. Fe_2_O_3_ shows an absorption band edges up to 580 nm. The wide absorption band of Fe_2_O_3_ in the visible region is due to the direct transition (O^2−^2p→Fe^3+^3d) and the spin-forbidden-excitations (Fe^3+^3d→3d), which rises the indirect transitions [36,37,38].

For the Fe_2_O_3_/zeolite, Figure 5C, the right edge of the photons uptake band shifts to a longer λ compared with that of Fe_2_O_3_, Figure 5B. This is correlated with the size of the nanoparticles of the Fe_2_O_3_ formed in the zeolite matrix. Hence, a broad and intense visible absorption range was observed for the Fe_2_O_3_/zeolite in Figure 5B. This would be better to achieve a massive electron–hole pair generation through electron transportation between the valence and conduction bands.

The absorbance values at λ = 500 nm are 0.185, 0.765 and 1.219 for zeolite, Fe_2_O_3_, and Fe_2_O_3_/zeolite, respectively, as seen in Figure 5D. This means more photons in the visible region, the concentrated portion of the solar light, can be absorbed by Fe_2_O_3_/zeolite than Fe_2_O_3_. This high absorbance refers to the dispersion of the Fe_2_O_3_ aggregates within the zeolite mesoporous structure and the modification of the electronic structure of Fe_2_O_3_/zeolite. Hence, zeolite has effectively enhanced the visible light absorption capability of the loaded Fe_2_O_3_ nanostructures. From Figure 5D, the general behavior of the transmittance spectrum of zeolite is the increase of transmittance% with the wavelength from UV to the visible region. The low transmittance for zeolite in the UV region is due to the existence of a strong absorption band in this region. The transmittance spectra for Fe_2_O_3_ and Fe_2_O_3_/zeolite (Figure 5D) can be divided into two regions. At wavelengths from 250 to 550 nm, the transmittance is nearly constant below 12%. Above 550 nm, the transmittance of Fe_2_O_3_ and Fe_2_O_3_/zeolite varies linearly with wavelength. The transmission of Fe_2_O_3_/zeolite is higher than that of Fe_2_O_3_ in the whole range of wavelengths.

The diffuse reflectance spectra (DRS) of the photocatalysts were measured to estimate the bandgap energies of the Fe_2_O_3_ and Fe_2_O_3_/zeolite. For this purpose, the Kubelka–Munk (K–M) model was used. Based on the following equation, this approach allows the absorption coefficient to be calculated by measuring diffuse light reflectance from a powdered mixture comprising absorbing and scattering components [39].

**Figure 6:**
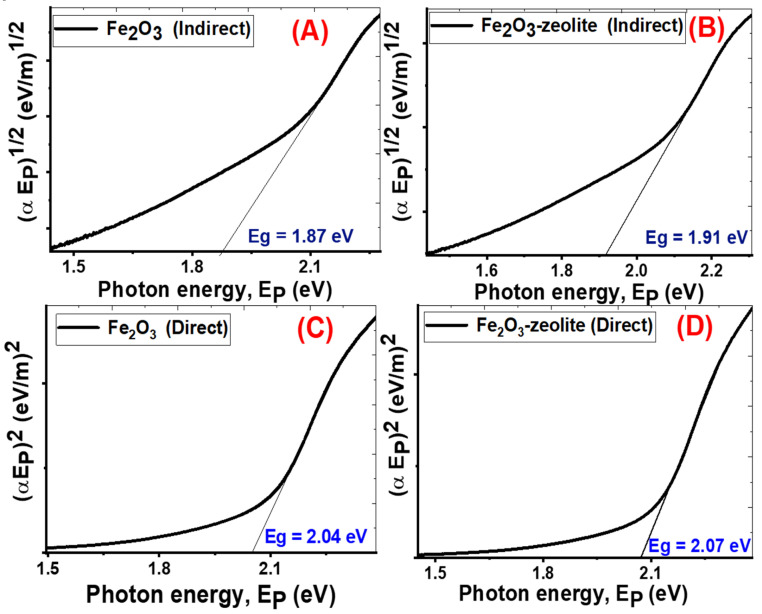
Indirect energy gap (**A**,**B**) for Fe_2_O_3,_ and Fe_2_O_3_/zeolite and direct energy gap (**C**,**D**) for Fe_2_O_3,_ and Fe_2_O_3_/zeolite, respectively.

F(R) = (1 − R)^2^/2R = α/S (1)

where F(R), R, S, and α indicate the K–M function, diffuse reflectance of the sample, the scattering coefficient, and the absorption coefficient, respectively. The K–M function is directly proportional to the absorption coefficient. Therefore, the direct and/or indirect band gaps of Fe_2_O_3_ and Fe_2_O_3_/zeolite were estimated by the following equation

(2) $$(\alpha \;E_{p}{)}^{n}\;=\;G\;(h\nu -\mathrm{Eg})$$

where $E_{p}$, Eg, and G refer to the photon energy, bandgap energy, and independent constant. For indirect bandgaps, n = 1/2, while for direct bandgaps, n = 2 [40]. The absorption bandgaps energies (direct or indirect) can be calculated from the straight-line portions of (${\alpha \;E}_{p}$)^n^ versus $E_{p}$ curve that intersects the energy axis, as shown in Figure 6.

The Fe_2_O_3_ and Fe_2_O_3_/zeolite have indirect bandgap values of 1.87 and 1.91 eV and direct bandgap values of 2.04 and 2.07 eV, respectively (Figure 6), which demonstrates the formation and incorporation of Fe_2_O_3_ nanoparticles in the zeolite. These values are consistent with the reported values for Fe_2_O_3_ prepared by different techniques in the [22,41]. Based on the quantization effect, the bandgap is proportional inversely to the crystallite size due to the confinement of the movement of electrons. Therefore, the increase in the bandgap of Fe_2_O_3_/zeolite compared to Fe_2_O_3_ can be understood based on the decrease in the crystallite size as seen in XRD data. This behavior is similar to that reported for many nanomaterials such as ZnO and ITO [42,43]. The studied optical properties suggest that the produced Fe_2_O_3_ from the rusted iron and its loading on zeolite as a host can greatly improve its semiconducting performance toward the massive absorption of the visible light. This suggests that the prepared Fe_2_O_3_/zeolite can be used for solar energy applications.

#### 3.1.5. FT-IR Study

FT-IR data of Fe_2_O_3_, zeolite and Fe_2_O_3_/zeolite nanocomposite are shown in Figure S1 (Supplementary Materials). The FT-IR spectrum of Fe_2_O_3_ nanoparticles was observed in the 4000–400 cm^−1^ wavenumber range, Figure S1. The bands of Fe_2_O_3_ appear at 1641 and 3415 cm^−1^, owing to the bending vibrations of the absorbed H_2_O and surface hydroxyl, and O–H stretch modes [20]. The appeared absorption modes at 2920 and 2850 cm^−1^ are assigned to the symmetric and asymmetric −CH_2_^−^groups stretch modes. A strong Fe–O asymmetric stretching mode was detected around 1040 cm^−1^ [44]. The located bands at 461, 537, and 790 cm^−1^ were attributed to the Fe–O stretch mode of Fe_2_O_3_ as confirmed in the literature [45]. A strong Fe–O asymmetric stretching mode was detected around 1040 cm^−1^ [44]. The located bands at 461, 537, and 790 cm^−1^ were attributed to the Fe−O stretch mode of Fe_2_O_3_ [45]. For zeolite, the bands at 3620 and 3446 cm^−1^ were attributable to Si–OH groups with H-bonding. The absorption mode at 1640 cm^−1^ was attributed to the OH bending mode [46]. The strong 1040, 790, and 600 cm^−1^ modes were significant to the internal asymmetric stretch and external symmetric stretch of X–O–X (X = Al or Si), and the internal X–O bending mode of AlO_4_/SiO_4_ tetrahedral [46]. The modes at 600 and 470 cm^−1^ authorize the existence of double five-membered rings of the pentasil zeolite [46]. For Fe_2_O_3_/zeolite, there are mixed bands between Fe_2_O_3_ and zeolite. The presence of broadband at 3429 cm^−1^ can be certified the O–H stretch mode, while the mode at 1650 cm^−1^ can be referred to as the O–H bending [47]. Bands of the zeolite appear at 1000 cm^−1^ in the nanocomposite, and the shift of these bands relative to that of zeolite refers to the break of H-bonds as a result of the existence of Fe on zeolite SiO_4_/AlO_4_ surfaces. Strong bands at 720, 598, 530, and 460 cm^−1^ were attributed to the symmetric vibration of (Al or Si)–O due to the internal vibration of zeolite.

### 3.2. Photoelectrocatalytic (PEC) H_2_ Generation

#### 3.2.1. PEC Characteristics and Conversion Efficiencies

PEC technology for converting solar energy to hydrogen via the water-splitting cycle was aided by the catalysts Fe_2_O_3_ and Fe_2_O_3_/zeolite. When Fe_2_O_3_ is subjected to light, the electron (e^−^) can be excited from the valence band, leaving a hole (h^+^) to the conduction band. The rate of hydrogen production depends on the lifetime of the carrier charge. The limitations of bare α-Fe_2_O_3_ faces in use as a PEC photoanode arise from the electronic structure of the material. The α-Fe_2_O_3_ suffers from a high density of mid bandgap trap states arising from closely spaced d levels that result in closely spaced optical transitions spanning the visible and into the near-ultraviolet regions. This leads to low carriers’ mobility and short lifetimes. In the Fe_2_O_3_/zeolite nanocomposite, the electrons can be trapped on the surface of the mesoporous zeolite. The zeolitic network can inhibit recombination of e/h pairs due to strong electric field strength through the distribution of photogenerated electrons inside zeolite [48]. Hence, the effective e^−^/h^+^ separation occur over robust interfacial interactions in Fe_2_O_3_/zeolite. This causes a decrease in e^−^/h^+^ recombination rates, which results in an efficient photoelectrocatalytic performance of Fe_2_O_3_-zeolite. Additionally, the Fe_2_O_3_/zeolite has a large effective surface area due to the porous framework of zeolite, which can increase PEC efficiency and allow for more intense absorption of incident light.

The optimized content of Fe_2_O_3_ and zeolite is highly desirable to reach high PEC performance. The photocurrent density is measured for Fe_2_O_3_ (III), Fe_2_O_3_ (II), and Fe_2_O_3_ (I) at an applied voltage of 1 V in 0.9 M KOH under light illumination, as seen in Figure S2 (Supplementary Materials). The photocurrent density is found to be 57.5, 48.82, and 42.64 mA/cm^2^ Fe_2_O_3_ (III), Fe_2_O_3_ (II), and Fe_2_O_3_ (I), respectively. Therefore, Fe_2_O_3_ (III) photoelectrode produces the highest photocurrent, which considers the optimized PEC photoelectrode. Additionally, nanocomposites of varied Fe_2_O_3_ (III)/zeolite weight ratios (0.2/1.8, 0.6/1.4, 0.8/1.2, 1.0/1.0, 1.2/0.8, 1.4/0.6, and 1.8/0.2) are utilized to manufacture Fe_2_O_3_/zeolite photoelectrodes for hydrogen production in order to optimize the nanocomposite composition. The photocurrent densities for all electrodes are measured under light illumination and at 1 V, as seen in Figure S3 (Supplementary Materials). The highest photocurrent density is found to be 57.93 mA/cm^2^ for Fe_2_O_3_/zeolite with a weight ratio of 1:1.

**Figure 7:**
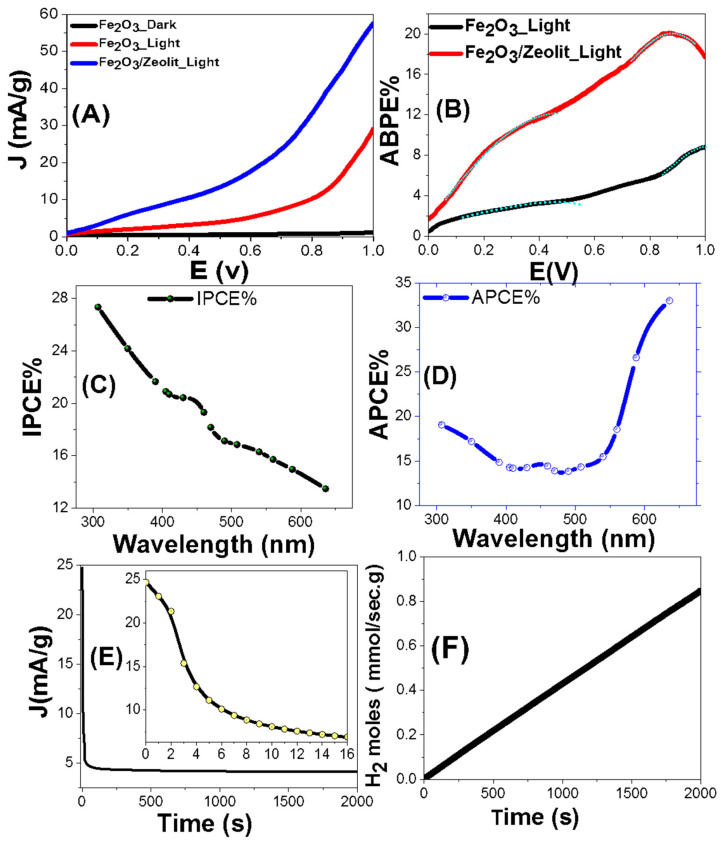
(**A**) The current density (J) vs. the applied potential for Fe_2_O_3_ and Fe_2_O_3_/zeolite under darkness and white light exposure, (**B**) APBE%, (**C**) IPCE%(λ), and (**D**) APCE%(λ)@1V vs. the incident wavelengths; variation of (**E**) J and (**F**) the number of H_2_ moles versus the exposure time.

Figure 7 shows the PEC performance of the optimized electrode. The variation of the current density (J) in darkness and white lighting from a metal-halide lamp versus the applied voltage (E) is presented in Figure 7A at 25 °C with a sweep rate of 0.1 mV/s. Using the Fe_2_O_3_ and Fe_2_O_3_/zeolite photo-electrocatalysts and in white lighting, the value of J is greatly enhanced vs. the positive applied voltage. By switching from the dark status to white light illumination status, the current density of Fe_2_O_3_ is increased from 1.14 to 29.1 mA/cm^2^ at +1 V, which refers to the PEC effect of Fe_2_O_3_. As shown in Figure 7A, J is increased by loading Fe_2_O_3_ on zeolite from 29.1 to 57.6 mA/cm^2^ at +1 V. This is due to the extending of the bandgap to the Vis/NIR range, which speeds up the redox reactions and then facilitates the PEC reaction. This also suggests a ~2-fold enhancement of the J-value relative to the Fe_2_O_3_ photocatalyst, which agrees with the increase of the surface charge, the extension of E_g_, and the strong absorptions in the Vis/NIR because of the loading of Fe_2_O_3_. In addition, it is very well-associated with the size variation of the Fe_2_O_3_ nanoparticles. Reduction in the size of Fe_2_O_3_ nanoparticles after loading on zeolite compared to Fe_2_O_3_ nanopowder, Figure 3, leads to greater surface areas and enhanced active surface spots that improve hydrogen generation activity. Additionally, Fe_2_O_3_/zeolite’s quantum confinement raises the reduction potentials to transfer the bound protons to H_2_ molecules. The quantum containment of Fe_2_O_3_/zeolite allows for further effective absorption in the Vis/NIR region (Figure 5). Note that Fe_2_O_3_ and Fe_2_O_3_/zeolite photoelectrocatalysts exhibit light-harvesting with J-values of 0.58 and 1.01 mA/cm^2^ at 0 V, and photocurrent onset at −0.098 and −0.056 V, correspondingly. It shows that, after loading Fe_2_O_3_ on the zeolite matrix, the interfacial transport resistances decrease, emphasizing the importance of the loading process in improving PEC efficiency. As a result of ions’ exchange ability, vacant sites in the zeolite surface also photoassisted hydrogen production [49]. Simultaneously, zeolite’s aluminosilicate frame is contributing to delayed charge carriers’ separations [50]. Since the control processes of electron/hole transfer are very important in photocatalytic reactions, zeolite can play an active role in electron transfer processes as an electron acceptor or electron donor. [51,52]. The Z-scheme mechanism for the nanocomposite can maintain photogenerated charge carriers with strong redox ability. The spatial isolation of charge carriers is providing a large driving force for the photocatalytic water reduction reaction [53]. To assess the photoelectrocatalysts’ performances as a tiny outer voltage is introduced between the electrodes of the PEC cell, the electrical energies introduced to the cell have to be deducted. This may be accomplished using the applied bias photon to current conversion efficiency (ABPE). The following Equation (3) is used to compute ABPE [54]:

(3) $$\mathrm{ABPE}(\%)=J\;\frac{\left(1.23-E_{\mathrm{app}}\right)}{p}\times 100$$

where E_app_ is the externally applied bias and p refers to the illuminating light power density (75 mW/cm^2^). Figure 7B demonstrates how ABPE varies with applied voltage at various wavelengths. The two highest ABPE% values are de-convoluted under white light illumination; (3.37% at 0.464 V and at 8.78% at 0.997 V) for Fe_2_O_3_ and (12.05% at 0.430 V and 20.01% at 0.882 V) for Fe_2_O_3_/zeolite. This indicates a ~3-fold improvement along with a decrease of the applied voltage, which can be beneficial for PEC cell operation. Additionally, Fe_2_O_3_/zeolite photocatalyst displays ABPE% of 1.64%at 0 V. This demonstrates that interfacial transport resistances have been reduced and photocatalytic performance has improved [54].

The enhanced solar absorption of the Fe_2_O_3_/zeolite photocatalyst is verified by estimating the photon-to-current incident efficiency (IPCE) at various wavelengths (λ) of the incident photons and constant potential (+1 V). The IPCE is calculated using the following Equation (4) [40]:

(4) $$\mathrm{IPCE}\%=1240.\frac{J}{\lambda .P}\;100\;$$

where λ is in nm. The variation of IPCE% with the wavelength of the monochromatic light for Fe_2_O_3_/zeolite photocatalyst is represented in Figure 7C. The highest IPCE% is ~27.34% @307 nm, in addition to another peak of 20.37% centered at ~440 nm corresponding to the highest absorption seen in Figure 5.

In the IPCE calculations, optical losses including transmittance (T) or reflectance (R) of incident photons were neglected. To compensate the optical losses, the absorbed photon to current conversion efficiency (APCE) is measured. APCE represents the number of photogenerated carriers that participate per absorbed photon in the generated photocurrent. The APCE is computed using the following Equation (5) [55]:

(5) $$\mathrm{APCE}(\lambda )=\frac{\mathrm{IPCE}\left(\lambda \right)}{A\left(\lambda \right)}=\frac{\mathrm{IPCE}\left(\lambda \right)}{1-R-T}$$

Here, A represents the optical absorbance. Figure 7D displays the behavior of APCE% as a function of the wavelength. As noted, APCE% is 19.1%@307 nm; then, it decreases to reach 13.8%@490 nm, followed by a successive increase to reach a maximum value of 33.0%@636 nm.

The stability of the Fe_2_O_3_/zeolite photocatalyst, for H_2_ generation, is studied for a prolonged time in 0.9 M KOH under white light and an applied voltage of +1 V Figure 7E shows the evolution of the J throughout time. The J-value dropped dramatically within the first 16 s, reaching roughly 6.9 mA/g. Then, limited photocorrosion processes occur between the PEC catalyst and the redox electrolyte, which account for the dramatic fall in the J-value [3]. For time > 16 s, before achieving a steady value of roughly 4.63 mA/g for 60 s, there is a slight reduction in J-value. This demonstrates that, in spite of the early decline in J-value, the Fe_2_O_3_/zeolite photocatalyst has high photochemical stability and a long lifespan as an active photocatalyst for the PEC H_2_ generation.

The full amount of hydrogen energy generated to the overall input sunlight energy (AM 1.5 G, 100 mW/cm^2^) is the solar-to-hydrogen conversion efficiency (STH). It can be used to calculate the total efficiency of the PEC cell [56]:

(6) $$\mathrm{STH}=[(\mathrm{mmol}\;H_{2}/s)\times \left(237\;\mathrm{KJ}/\mathrm{mol})]/[P_{\mathrm{total}}\times \mathrm{ECSA}\right]$$

where P_total_, ECSA, and H_2_/S refer to the total light power density in mW cm^−2^, the electrochemical surface area in cm^2^, and the rate of hydrogen generation/s, respectively. Applying Faraday’s law, the number of generated H_2_ moles by the PEC cell can be calculated using Equation (7).

(7) $$H_{2}(\mathrm{moles})=\int_{0}^{t}\frac{\mathrm{Jdt}}{F}$$

Here, F refers to the Faraday constant (9.65 × 10^4^ C/mol), and t is the period of generation. Figure 7F shows the variation of H_2_ (moles) versus the production time. The creation rate of H_2_ is 154.44 mmol h^−1^ g^−1^. Zeolite plays an effective role in the rapid spread of hydrogen bubbles which escape from the photocatalyst. This paves the way for higher current and additional H_2_ creation over the same period [57].

The ECSA of Fe_2_O_3_ and Fe_2_O_3_/zeolite photocatalysts were obtained utilizing the Randles–Sevcik equation,

ECSA= I(RT)^0.5^ (C n F)^−1.5^(*v* D)^−0.5^/0.4463 (8)

Here, n, C, and T stand for the number of electrons in a redox reaction (n = 1), analyte concentration, and temperature, correspondingly, while F, R, and D stand for Faraday, gas-molar, and analyte diffusion constants [26]. Utilizing Figure 7A, the ECSAs of the photocatalysts were found using ECSA = Q·m^−1^/C, whereas Q, m, and C indicate the negative-scan hydrogen-adsorption charges after double-layer charge modification, photocatalyst mass, and complete monolayer charges of the electrode-cover H-atoms, respectively [26]. The Q value was estimated by integrating the curve of the photocatalyst, Figure 7A, divided by the scan rate. The values of ECSA for the photocatalysts are determined and presented in Table 3. For Fe_2_O_3_ and Fe_2_O_3_/zeolite, the values were 7.414 and 21.236 m^2^/g, respectively. The 3-fold improvement in the ECSA explains the improved PEC performance, Figure 7A, of Fe_2_O_3_/zeolite photocatalyst versus the Fe_2_O_3_. Then, the estimated STH value was 12.74% for the Fe_2_O_3_/zeolite photocatalyst.

#### 3.2.2. Corrosion and Tafel Parameters of Fe_2_O_3_ and Fe_2_O_3_/Zeolite Photocatalysts

**Figure 8:**
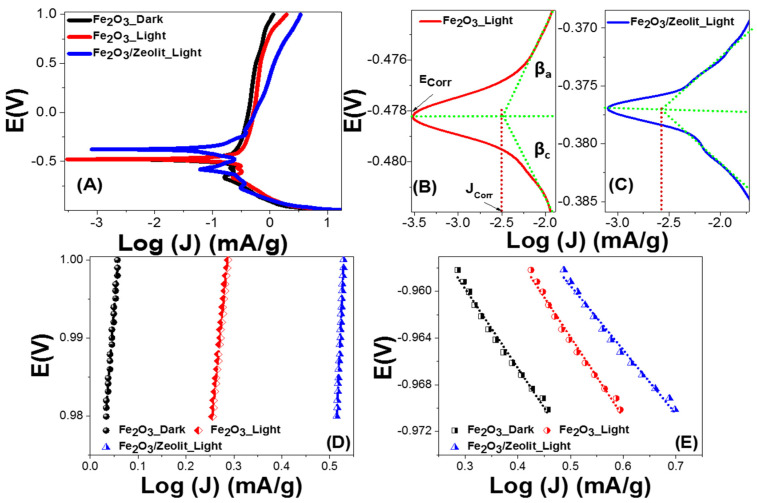
Combined anodic and cathodic polarization of Fe_2_O_3_ and Fe_2_O_3_/zeolite (**A**), E_corr_ and J_corr_ of (**B**) Fe_2_O_3_ and (**C**) Fe_2_O_3_/zeolite; calculation of (**D**) anodic (*β*_a_) and (**E**) cathodic (*β*_c_) Tafel slopes.

The Tafel relationship, V = β log(J) + C, was used to quantify combined anodic and cathodic Tafel or polarization parameters to determine the mechanism of the H_2_ generation reaction(HGR) and the rate-limiting phase [58]. Low Tafel slopes, high current exchange rates, and good HGR performances are all characteristics of the ideal photocatalyst. Figure 8A shows the Tafel plots for Fe_2_O_3_ and Fe_2_O_3_/zeolite. Figure 8B,C displays the main characteristics: corrosion potential and current (E_corr_ and I_corr_) and anodic (*β*_a_) and cathodic (*β*_c_) Tafel slopes for the Fe_2_O_3_ and Fe_2_O_3_/zeolite. The values of *β*_a_ and *β*_c_ for Fe_2_O_3_ and Fe_2_O_3_/zeolite are found using the slopes of the curves’ linear segments, as shown in Figure 8D,E [26,59]. The obtained values of E_corr_, I_corr_, *β*_a_, and *β*_c_ were presented in Table 3 for Fe_2_O_3_ and Fe_2_O_3_/zeolite. For Fe_2_O_3_/ zeolite, the *β*_a_ and *β*_c_ values are 139.9 and 5.5 mV dec^−1^, respectively, and 63.4 and 6.8 mV dec^−1^ for Fe_2_O_3_. The PEC HGR mechanism and rate-limiting phases are indicated by the Tafel slopes. The Volmer–Tafel mechanism is predominant when the recombination phase is a rate limit and the Tafel slope is 30 mV dec^−1^. The Volmer–Heyrovsky H_2_ generation process could be presumed to be dominant when PEC desorption is a rate limit and the Tafel slope is 40 mV dec^−1^. The reaction pathways are dependent on the surfaces covered with adsorbed hydrogen if the Tafel slope is 120 mV dec^−1^. The *β*_c_-value denotes the needed over-potential to enhance the HGR rate by a factor of ten [26,59]. The low values of βc refer to the low optical band gaps of the designed Fe_2_O_3_ and Fe_2_O_3_/zeolite photocatalysts. This means that small amounts of energy (low overpotentials) are needed to achieve efficient HGR.

The corrosion rate is directly dependent on I_corr_, where E_corr_ offers aspects about the solution’s corrosion propensity. From Figure 8A–C, the Fe_2_O_3_/zeolite presents nobler behavior. The Fe_2_O_3_/zeolite has a smaller E_corr_ (376.7 mV) than Fe_2_O_3_ (478.3 mV). Generally, the E_corr_ values revealed in this work are greater than any earlier stated values for Fe_2_O_3_-based photocatalysts and are moved to more noble behaviors when compared to commercial Fe_2_O_3_ [60].

To verify the relative ability of the electrode to resist corrosions; the values of I_corr_, polarization resistance (R_P_), and corrosion rate (CR) could be determined. The CR is related to the kinetic value I_corr_ directly, while R_p_ is inversely proportional. From Table 2, the loading of Fe_2_O_3_ on the zeolite host reduces I_corr_ from 3.15 to 2.66 μA cm^−2^, which is much smaller than any previously reported Fe_2_O_3_ photoelectrode’s corrosion current. For example, Kim et al. reported 5.31 μA/cm^2^ for Fe_2_O_3_ and 8.69 μA/cm^2^ for Fe_3_O_4_ [60]. The values of R_p_ are determined by the Stern–Geary equation, R_p_ = *β*_a_ *β*_c_/[2.303 I_corr_ (*β*_a_ + *β*_c_)], utilizing the straight segments near to E_corr_ of the curves. The values of CR (n year^−1^) are determined by CR = 3272 [I_corr_ × W/(ECSA × d)], whereas EW and d represent the equivalent weight (g eq^−1^) and density (g cm^−3^). For Fe_2_O_3_ and Fe_2_O_3_/zeolite, the values of Rp and CR are reported in Table 3. The Rp values are increased from 847.66 to 864.98 Ω cm^2^, whereas CR is decreased from 15.02 to 7.61 pm Year^−1^ by loading Fe_2_O_3_ on zeolite host. Therefore, photocorrosion is suppressed by the loading of the Fe_2_O_3_ photocatalyst into zeolite [61]. This is because zeolite can provide specific photophysical properties such as preventing the Fe_2_O_3_ nanoparticles from aggregating and improving their stability against sinterisation. The above-mentioned corrosion metrics show a significant improvement of the Fe_2_O_3_ photocatalyst’s stability through the use of zeolite as catalyst support. The obtained CR values outperform any prior Fe_2_O_3_-based PEC electrode results [62,63].

## 4. Conclusions

A highly effective recycling technique for rusted iron wastes and a scalable method for the preparation of Fe_2_O_3_ and Fe_2_O_3_/zeolite nanocomposite have been reported. The Fe_2_O_3_/zeolite nanocomposite showed smaller sizes, more homogeneous nanopore diameter distribution, greater Vis/NIR light absorption capability, and a wider bandgap than Fe_2_O_3_ nanopowder. Fe_2_O_3_/zeolite nanocomposite was applied successfully as a low-cost nanophotocatalyst. The application of Fe_2_O_3_/zeolite for photoelectrocatalytic hydrogen production showed a production rate of 154.45 mmol g^−1^ h^−1^ at 1 V in 0.9 M KOH solution, which is the highest value yet for Fe_2_O_3_-based photocatalysts. The photocurrent density of Fe_2_O_3_/zeolite is almost 2-fold that of the Fe_2_O_3_ catalyst, and the IPCE% reached ~27.34%@307 nm and 1 V nm. This nanophotocatalyst has also shown remarkable stability with a very low PEC corrosion rate of 7.6 pm/year. Additionally, it can retain ~97% of its initial performance.

## Figures and Tables

**Table 1:** The ball milling conditions for preparing zeolite.

Condition	Description
Vessel size	15 cm
Diameter balls	from 1.11 to 1.75 cm
Materials of vessels	stainless steel
Materials of balls	porcelain
Ball/precipitate mass ratio	8:1 mass ratio
Speed	5000 rpm
Time	5 h

**Table 2:** Values of the crystallographic parameters of Fe_2_O_3_ and Fe_2_O_3_/zeolite nanohybrid.

Parameter	Planes (hkl)	Position (° 2Th.) (degree)	Height (cts)	d-Spacing (Å)	Relative Intensity (%)	Crystallite Size (nm)	Microstrain (ε)	Dislocation (δ) (10^−4^ nm^−2^)
Fe_2_O_3_	(110)	35.39	83.37	2.536	89.78	50.46	0.251	3.927
	(104)	33	92.86	2.714	100	64.84	0.209	2.378
Fe_2_O_3_/zeolite	(110)	35.77	27.45	2.51	67.22	47.85	0.262	4.367
	(104)	33.32	40.83	2.689	100	56.53	0.238	3.129

**Table 3:** ECSA values and corrosion and Tafel parameters for Fe_2_O_3_ and Fe_2_O_3_/zeolite photocatalysts.

Sample	ECSA (m^2^/g)	E_Corr_ (mV)	I_Corr_ (μA cm^−2^)	*β*_a_ (mV dec^−1^)	R^2^	*β*_c_ (mV dec^−1^)	R^2^	R_p_ (Ω cm^2^)	Corr Rate (nm year^−1^)
Fe_2_O_3_	7.414	478.28	3.15	63.4 ± 0.9	0.996	6.8 ± 0.2	0.988	847.66	0.01502
Fe_2_O_3_/zeolite	21.236	376.72	2.66	139.9 ± 2.8	0.992	5.5 ± 0.1	0.989	864.98	0.00761

## Data Availability

Not applicable.

## Supplementary Materials

The following are available online at https://www.mdpi.com/article/10.3390/nano11123445/s1, Figure S1: FT-IR spectra of Fe_2_O_3_, zeolite, and Fe_2_O_3_/zeolite nanocomposite. Figure S2: Variation of current density (J) for Fe_2_O_3_ (I), (II), and (III) under white light illumination and at 1 V. Figure S3: Variation of current density (J) for Fe_2_O_3_ (III)/zeolite photoelectrodes with different Fe_2_O_3_ (III)/zeolite weight ratios at 1 V under white light illumination.
