# Supplementary material for: Recycling Rusty Iron with Natural Zeolite Heulandite to Create a Unique Nanocatalyst for Green Hydrogen Production
Source: Nanomaterials (Basel). 2021 Dec 20;11(12):3445. doi: 10.3390/nano11123445 (PMC8704551; doi:10.3390/nano11123445)
Supplement: Supplementary file 1 [file nanomaterials-11-03445-s001.zip › nanomaterials-1430649-supplementary.pdf]

Supplementary Materials

Recycling Rusty Iron with Natural Zeolite Heulandite to Create a Unique Nanocatalyst for Green Hydrogen Production

Mohamed Shaban [1,2,*], Mohammad BinSabt [3], Ashour M. Ahmed [2] and Fatma Mohamed [2,4]

1 Department of Physics, Faculty of Science, Islamic University in Madinah, Al-Madinah Al-Munawarah 42351, Saudi Arabia
2 Nanophotonics and Applications (NPA) Lab, Physics Department, Faculty of Science, Beni-Suef University, Beni-Suef 62514, Egypt; ashour.elshemey@gmail.com (A.M.A.); f_chem2010@yahoo.com (F.M.)
3 Chemistry Department, Faculty of Science, Kuwait University, P.O. Box 5969, Safat 13060, Kuwait;
Mohammad.binsabt@ku.edu.kw
4 Polymer Research Laboratory, Chemistry Department, Faculty of Science, Beni-Suef University,
Beni-Suef 62514, Egypt; f_chem2010@yahoo.com
* Correspondence: mssfadel@aucegypt.edu

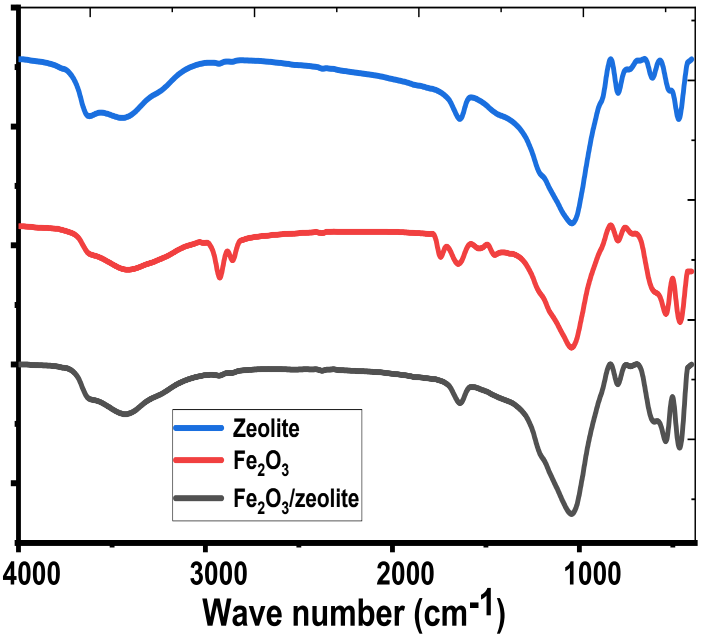

Figure S1. FT-IR spectra of Fe_2O_3, zeolite, and Fe_2O_3/zeolite nanocomposite.

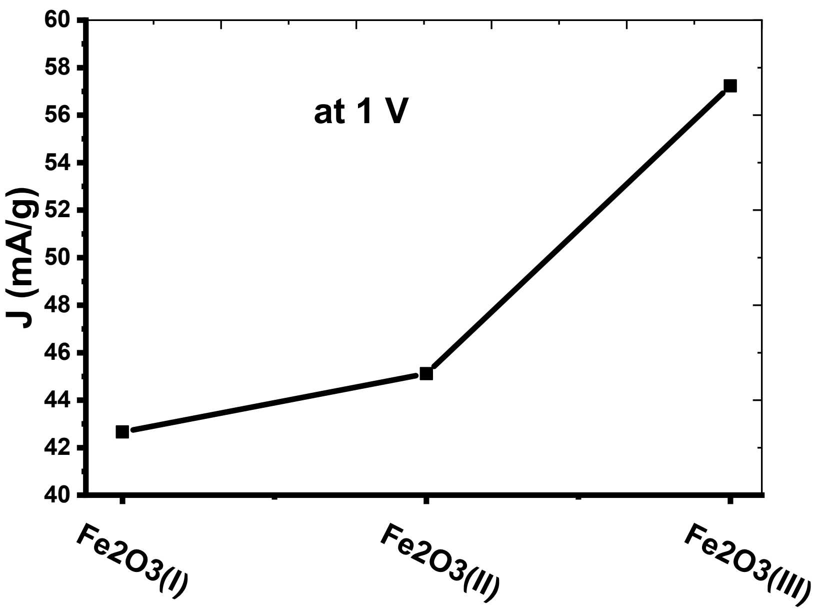

Figure S2. Variation of current density (J) for Fe_2O_3 (I), (II), and (III) under white light illumination and at 1 V

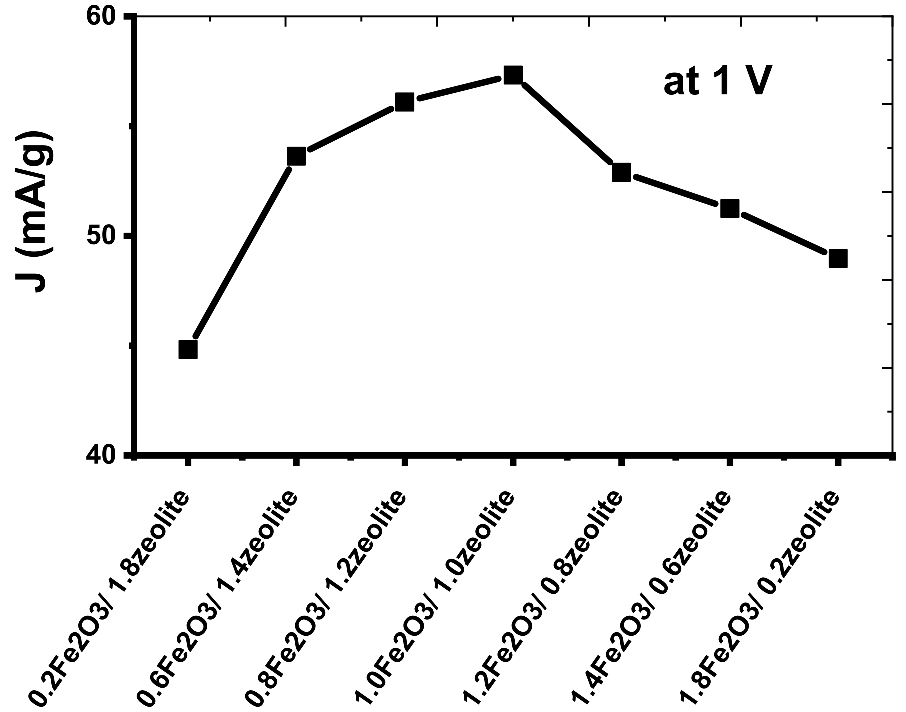

Figure S3. Variation of current density (J) for Fe_2O_3 (III)/zeolite photoelectrodes with different Fe_2O_3 (III)/zeolite weight ratios at 1 V under white light illumination
